# Erlotinib plus gemcitabine versus gemcitabine for pancreatic cancer: real-world analysis of Korean national database

**DOI:** 10.1186/s12885-016-2482-z

**Published:** 2016-07-11

**Authors:** Sangjin Shin, Chan Mi Park, Hanbyeol Kwon, Kyung-Hun Lee

**Affiliations:** National Evidence-based healthcare Collaborating Agency, Seoul, Korea; Department of Internal Medicine, Seoul National University Hospital, 101 Daehak-Ro, Jongno-Gu, Seoul, 110-744 South Korea; Cancer Research Institute, Seoul National University College of Medicine, Seoul, Korea

**Keywords:** Pancreatic cancer, Gemcitabine, Erlotinib, Comparative effectiveness research, National database

## Abstract

**Background:**

A randomized clinical trial has found that the addition of erlotinib to gemcitabine (GEM-E) for pancreatic cancer led to a modest increase in survival. The aim of this national population-based retrospective study was to compare the effectiveness of GEM-E to GEM alone for pancreatic cancer patients in real clinical practice.

**Methods:**

Patients with pancreatic cancer (ICD-10: C25) with prescription claims of gemcitabine or erlotinib between Jan 1, 2007 and Dec 31, 2012 were retrospectively identified from the Korean Health Insurance claims database. To be included in the study population, patients were required to have had a histological or cytological diagnosis within one year before chemotherapy. Patients treated with prior radiotherapy, surgery, or chemotherapy were excluded to reduce heterogeneity. Overall survival from the initiation of therapy and the medical costs of GEM-E and GEM were compared.

**Results:**

A total of 4,267 patients were included in the analysis. Overall survival was not significantly longer in patients treated with GEM-E (median 6.77 months for GEM-E *vs.* 6.68 months for GEM, *p* = 0.0977). There was also no significant difference in the respective one-year survival rates (27.0 % *vs.* 27.3 %; *p* = 0.5988). Multivariate analysis using age, gender, and comorbidities as covariates did not reveal any significant differences in survival. Based on this relative effectiveness, the incremental cost per life year gained over GEM was estimated at USD 70,843.64 for GEM-E.

**Conclusions:**

GEM-E for pancreatic cancer is not more effective than GEM in a real-world setting, and it does not provide reasonable cost-effectiveness over GEM.

**Electronic supplementary material:**

The online version of this article (doi:10.1186/s12885-016-2482-z) contains supplementary material, which is available to authorized users.

## Background

Pancreatic cancer is a major problem, causing 266,000 estimated deaths per year worldwide, with an estimated mortality-incidence ratio of 0.95 [1]. In South Korea, it currently ranks eighth in incidence and fifth in cancer-related mortality. The 5-year overall survival (OS) is less than 10 % [2]. At the time of diagnosis, approximately half the patients with pancreatic cancer have metastases, and their median survival does not exceed 6 months [3]. Despite efforts to improve therapeutic strategies, the 5-year OS has not increased significantly over the past decade [4, 5].

Gemcitabine (GEM) has provided survival superior to bolus 5-FU, and for more than a decade has been considered to be the standard treatment for metastatic pancreatic cancer [6, 7]. GEM-based combination regimens were subsequently evaluated for superiority over GEM alone in clinical trials [8, 9]. However, with the exception of erlotinib, the addition of targeted agents to GEM has failed to produce any added benefit [10, 11]. The combination of gemcitabine and erlotinib (GEM-E) showed a modest increase in survival (median survival 6.24 months *vs.* 5.91 months, 1-year survival increased to 23 % from 17 %, compared with GEM alone) [12, 13]. Although the improvement in survival provided by the combination was statistically significant, it is questionable whether the two-week improvement in survival is clinically meaningful. Moreover, an actual improvement in real clinical practice is also questionable, considering the difference between controlled trials and the real world.

Since the randomized phase III trial that showed improvement for GEM-E [12], it was approved by South Korean Food and Drug Administration as the first-line treatment for patients with advanced pancreatic cancer. GEM-E has been reimbursed by the Korean Ministry of Health and Welfare and the National Health Insurance Service (NHIS) since 2006. This reimbursement policy reflects the urgent need for more effective treatment for advanced pancreatic cancer. However, because of the concerns about the real-world efficacy of GEM-E, it has been proposed that there is a need for reassessment using a national database.

Use of the NHIS database to quantify treatment utilization and effectiveness of treatment in routine practice has several advantages. First, South Korea provides universal health insurance coverage. NHIS covers approximately 97 % of the entire 50.6 million South Korean population and is fairly representative of the Korean population. Second, NHIS includes data on the diagnosis, which is recorded according to the International Classification of Diseases, 10th revision (ICD-10); procedures; prescriptions (drug name, formula, dose, duration of prescription, costs); and demographics. With the NHIS database, it is possible to monitor from a payer perspective the impact of adopting new drugs on resource utilization and the effectiveness of new treatments used in routine clinical practice. These databases have been used in previous studies [14–18].

This retrospective study aimed to evaluate the effectiveness and cost effectiveness of GEM-E compared to GEM for pancreatic cancer patients, using data from the South Korean NHIS claims database.

## Methods

### Selection of patients with pancreatic cancer

This was a population-based, retrospective analysis using the NHIS database to identify patients with pancreatic cancer who began chemotherapy with GEM-E or GEM between January 1, 2007 and December 31, 2012.

The study population included patients who received GEM therapy by injection for pancreatic cancer (ICD-10: C25). Within study population, GEM-E patients were identified if erlotinib was prescribed with gemcitabine injection at index date. Patients were required to have a history of intervention for histological or cytological diagnosis within one year before the index date, because the final results of these evaluations were not available from the database. To avoid possible heterogeneity of the patients, patients initially presenting with metastatic pancreatic cancer treated with first-line chemotherapy with GEM or GEM-E were included in this study, excluding those who received prior radiotherapy or surgical treatment before GEM or GEM-E.

Patients with a history of receiving GEM before 2007 were excluded. We also excluded patients who had a diagnosis of hepatobiliary cancer (C24), bronchial and lung cancer (C34), breast cancer (C50), ovarian cancer (C56), and bladder cancer (C67), each within 5 years before and after the index date; those who were diagnosed with pancreatic neuroendocrine cancer (C25.4) after cohort entry; those who received GEM as a therapy for other cancers; and those who were younger than 18 years.

The Charlson Comorbidity Index (CCI) score was used to assess the overall burden of comorbidity [19]. Patients were followed for three years or until date of death or end of the study period (December 31, 2013), whichever came first.

Because the claims data that were used were fully deidentified, approval from the Institutional Review Board was not required for this study.

### Clinical and economic outcomes

The clinical outcome of the study was overall survival, which was calculated from the date of GEM or GEM-E to the date of death from any cause. Medical cost per patient for GEM-E and GEM was measured using claims data during the entire follow-up period. Total medical costs were calculated as the sum of medication costs, outpatient costs, and inpatient costs. All costs were calculated in Korean won (KRW) and converted into US dollars (USD) using the yearly average exchange rate for 2013 (average rate: 1 USD = 1,113.85 KRW) and the annual rate of adjustment for medical fees in the insurance scheme to reflect inflation during 2007 and 2013 [20].

### Statistical analysis

The two-sided t-test was used to make between-group comparisons of continuous data, and the chi-square test was used for categorical data. OS was calculated from the date of initiation of GEM-E or GEM to the date of last follow-up or death from any cause, using the Kaplan-Meier method and log-rank test. Multivariate analysis of survival was performed using the Cox proportional-hazards model to evaluate treatment effect, with adjustment for sex, age, and comorbidities. For the skewed distribution of cost, treatments were compared using the t-test after log transformation. All data manipulation and statistical analyses were performed using SAS 9.1.3 (SAS institute, Cary, NC, USA). A *p*-value <0.05 was considered statistically significance.

## Results

### Patient characteristics

**Fig. 1:**
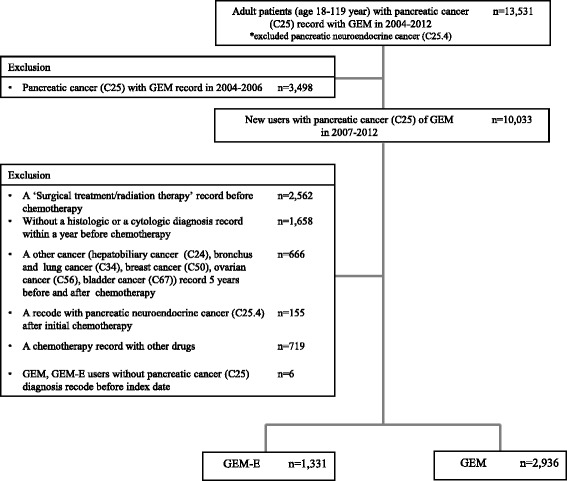
Selection of the study population. Abbreviation: GEM, Gemcitabine; GEM-E, Gemcitabine + Erlotinib

We identified 13,531 patients between January 1, 2004, and December 31, 2012. Among them, 3,498 patients who received chemotherapy between January 1, 2004, and December 31, 2006, were excluded. Additionally 2,562 patients who had had surgical treatment or radiotherapy prior to the index date were excluded. Also excluded were 1,658 patients without a history of intervention for histological or cytological diagnosis within one year before chemotherapy. Patients (*n* = 666) who had other cancers; and patients (*n* = 155) with at least one claim of pancreatic neuroendocrine cancer (ICD-10 code: C25.4) after the index date were excluded. Patients (*n* = 719) who were treated with GEM or GEM-E concurrently with other chemotherapeutic agents were excluded (Fig. 1).

Finally, a total of 4,267 patients who had chemotherapy with either GEM (*n* = 1,331, 31.2 %) or GEM-E (*n* = 2,936, 68.8 %) satisfied the inclusion criteria. The baseline characteristics of patients are summarized in Table 1. The mean ages ± standard deviation (SD) of the patients receiving GEM-E or GEM were 61.9 ± 9.8 years and 63.4 ± 10.4 years, respectively (*p* < 0.0001). There were significantly more male patients in the GEM-E group (64.7 % *vs*. 59.4 %, *p* = 0.0010). The mean CCI scores ± SD of the patients receiving GEM-E or GEM at the index date were 8.88 ± 3.39 and 9.14 ± 3.40, respectively (*p* = 0.0206).

**Table 1:** Patient baseline characteristics

	GEM-E (*n* = 1,331)	GEM (*n* = 2,936)	*p*-value^a^
	No. (%)	No. (%)	
Sex			
Male	861 (64.69)	1,743 (59.37)	0.0010
Female	470 (35.31)	1,193 (40.63)	
Age, years			
Mean ± SD	61.89 ± 9.76	63.38 ± 10.40	<0.0001
Median	63	65	
Range	28–88	18–92	
18–29	2 (0.15)	6 (0.20)	<0.0001
30–39	16 (1.20)	42 (1.43)	
40–49	128 (9.62)	268 (9.13)	
50–59	387 (29.08)	683 (23.26)	
60–69	486 (36.51)	1,010 (34.4)	
70–79	295 (22.16)	825 (28.1)	
Above 80	17 (1.28)	102 (3.47)	
Charlson Comorbidity Index (CCI)			
Mean ± SD	8.88 ± 3.39	9.14 ± 3.40	0.0206
Median	10	10	
Range	2–18	2–19	
Comorbidity score			
≤ 3	102 (7.66)	222 (7.56)	0.2024
4–6	246 (18.48)	476 (16.21)	
7–9	302 (22.69)	664 (22.62)	
10–12	511 (38.39)	1,134 (38.62)	
≥ 13	170 (12.77)	440 (14.99)	
Follow-up time, days^b^			
Mean ± SD	278.7 ± 224.7	292.3 ± 263.3	
Median	210	207	

Abbreviation: *GEM* Gemcitabine, *GEM-E* Gemcitabine + Erlotinib

^a^Differences between GEM and GME-E were tested with t-test for continues variable and with the chi-square test for categorical variables

^b^Follow-up termination: follow-up for 3 years or until December 31, 2013 or date of death

**Table 2:** Summary of Effectiveness

	GEM-E	GEM	*p*-value^a^
	(*n* = 1,331)	(*n* = 2,936)	
Overall survival time, months			
Mean ± SD	10.48	9.86	0.0977
	(325.5 days ± 8.9)	(305.6 days ± 5.4)	
Median	6.77	6.68	
	(210 days)	(207 days)	
Overall survival rate per treatment time, %			
6 months	56.5	54.7	0.2108
12 months	27.0	27.3	0.5988
24 months	12.9	10.6	0.1350
36 months	9.3	6.5	0.0977

Abbreviation: *GEM* Gemcitabine, *GEM-E* Gemcitabine + Erlotinib

^a^log-rank test

### Clinical outcome

**Fig. 2:**
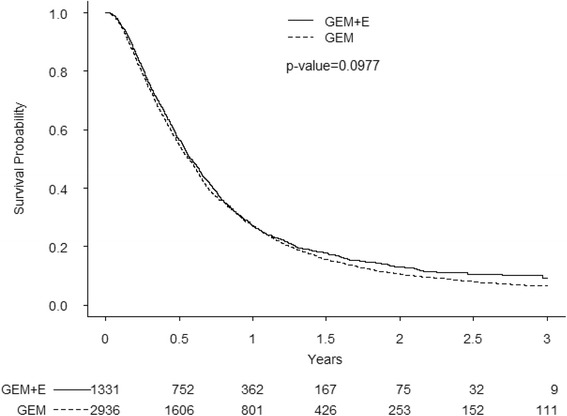
Kaplan-Meier curves for overall survival. Abbreviation: GEM, Gemcitabine; GEM-E, Gemcitabine + Erlotinib

The survival curves for each treatment, derived from the Kaplan-Meier estimates, are shown in Fig. 2. The median overall survival of the patients receiving GEM-E was not significantly longer (6.77 months for GEM-E *vs.* 6.68 months for GEM, *p* = 0.0977) (Table 2). The actual difference in median survival was 3 days (mean 20 days). The 1-year survival rates of patients receiving GEM-E and GEM were 27.0 and 27.3 %, respectively (*p* = 0.5988). Multivariate analysis using age, sex, and comorbidities as covariates did not reveal any significant differences in survival (hazard ratio 0.966, *p* = 0.3353, Additional file 1: Table S1). For sensitivity analysis, the follow-up period was extended for five years; however, the median overall survival time was similar to the survival seen in the base analysis (data not shown).

### Cost-effectiveness results

The total medical costs associated with treatment were USD 3,891.53 higher per patient receiving GEM-E (USD 23,819) than the costs of those receiving GEM (USD 19,927; Table 3). The mean medication costs of the patients receiving GEM-E were significantly higher than the costs of the patients receiving GEM group, reflecting the cost of erlotinib (USD 3,264.00 vs. USD 1,169.20, respectively; *p* < 0.0001; Table 3).

**Table 3:** Medical cost estimation

(Unit: USD)			
	GEM-E	GEM	*p*-value^a^
	(*n* = 1,331)	(*n* = 2,936)	
Inpatient cost (Mean ± SD)^b^	14,846.70 ± 11,865.27	13,211.07 ± 1,0931.57	<0.0001
Length of stay (Mean ± SD)	64 ± 53	63 ± 54	0.8032
Outpatient cost (Mean ± SD)^b^	6,152.29 ± 7,233.05	6,066.95 ± 7,001.03	0.6920
Medication cost (Mean ± SD)^b^	3,264.00 ± 4,579.44	1,169.20 ± 1,644.40	<0.0001
Total medical costs (Mean ± SD)^b^	23,819.51 ± 15,106.18	19,927.98 ± 14,444.86	<0.0001

Abbreviation: *GEM* Gemcitabine, *GEM-E* Gemcitabine + Erlotinib

^a^Differences between GEM and GEM-E was tested with t-test after log transformation

^b^Average 2013 exchange rate: one US dollar (USD) = 1,113.85 Korea Won (KRW)

The average and incremental cost-effectiveness ratios derived from the total costs and the outcome measures are shown in Table 4. Expressed as a cost-effectiveness ratio for an additional survival of 20 days, the incremental cost per life year of GEM-E over GEM was estimated to be USD 70,843.64. GEM-E was not cost-effective at the Korean willingness-to-pay (WTP) threshold of USD 27,272 [21].

**Table 4:** Cost-Effectiveness analysis results

	GEM-E	GEM
	(*n* = 1,331)	(*n* = 2,936)
Effectiveness	325.6 days	305.6 days
(Overall mean survival time)	(0.894 year)	(0.839 year)
Incremental Effectiveness	20 days	-
	(0.055 year)	
Cost^a^	USD 23,819.51	USD 19,927.98
Incremental Cost	USD 3,891.53	-
Incremental Cost-Effectiveness Ratio (ICER)	USD 70,755.06/year	-

Abbreviation: *GEM* Gemcitabine, *GEM-E* Gemcitabine + Erlotinib

^a^Average 2013 exchange rate: one US dollar (USD) = 1,113.85 Korea Won (KRW)

## Discussion

GEM has been the standard-of-care for patients with advanced or metastatic pancreatic cancer [6], and the addition of other chemotherapeutic agents to GEM had failed to show superiority over GEM alone [10, 11] until the positive result for abraxane [22]. The addition of erlotinib to GEM has been shown to be superior to GEM alone with statistical significance [12, 13], but using GEM-E instead of GEM for the modest survival difference of 2 weeks has been a matter of debate. Moreover, there have not been additional clinical trials that have directly compared GEM and GEM-E for advanced or metastatic pancreatic cancer, and the data regarding GEM-E is thus limited.

This study investigated the effectiveness of GEM-E over GEM alone using a national population-based claims database, and we could not find significant differences between the treatments. This is the first study, to our knowledge, that has investigated the real-world effectiveness of GEM-E versus GEM.

Although the patients receiving GEM-E were younger (61.9 vs 63.4 years), had lower CCI scores (8.9 vs 9.1), and there were more males (64.7 % vs 59.4 %), compared with the patients receiving GEM; there was no survival advantage for the patients receiving GEM-E. Because a higher CCI score was associated with worse survival (Additional file 1: Table S1), it is plausible that the baseline characteristics of the patients receiving GEM were not favorable. However, the final outcomes, as reflected in overall survival, were not significantly different.

There are some differences between our national, unselected, large study cohort in our retrospective observational study and the patients enrolled in the randomized controlled trial (RCT) conducted by Moore et al. [12] RCTs are an effective method for determining and validating the efficacy of a treatment, and the remarkable improvement in the treatment of cancer over the past few decades can be attributed to RCTs [23, 24]. Despite the excellent internal validity of RCTs, however, the external validity and generalizability of RCTs may be limited; and data from independent patient groups are often required to confirm RCT results. Population-based observational research can provide data with good external validity and can complement the limitations of RCTs. As the superiority of GEM-E over GEM alone was shown by only one RCT [12], our analysis of the real-world effectiveness of GEM-E is of value.

South Korea has universal health insurance that covers virtually the entire population, and this study extracted and screened all the patients with pancreatic cancer from South Korea’s NHIS database. The study is representative of all the patients in South Korea with advanced pancreatic cancer between 2007 and 2012, and can provide good external validity. Moreover, comorbidity data, as well as patient demographics and claims for medical services submitted to the national insurance system, were retrieved from the electronic database; and these data also contribute to the strength of the external validity.

Limitations inherent to population-based studies and the claims-based approach affect this study. The diagnosis code on a claim does not necessarily verify the specific disease, since the diagnosis may be incorrectly coded or a code may not precisely capture the diagnosis of interest. The inclusion criterion of this study that each patient must have received a histological or cytological diagnosis within one year before the index date probably reduced the sensitivity, but increased the specificity identification of patients with pancreatic cancer. Also, information that could have affected the study outcomes, such as performance status is not readily available from claims data. The results of our study, thus, should be interpreted with caution; however, this is the first and the largest study to compare effectiveness of first chemotherapy for pancreatic cancer reflecting the real-world clinical benefit.

Since the introduction of GEM-E as one of the standard-of-care treatments for advanced pancreatic cancer, other regimens also have shown superiority over GEM alone. FOLFIRINOX (5-FU, leucovorin, irinotecan, and oxaliplatin) first showed superiority over GEM with better response rate (32 versus 9 %), longer progression-free survival (6.4 versus 3.3 months), and longer survival (11.1 versus 6.8 months) [25]. The addition of albumin-bound (nab)-paclitaxel to GEM also resulted in better response rate (23 versus 7 %), longer progression-free survival (5.5 versus 3.7 months), and longer survival (8.5 versus 6.7 months) [22]. The magnitude of benefit from these regimens, shown by the survival differences, are absolutely larger than the benefit of GEM-E, and these combination regimens are recommended in patients with good performance status. Further research on the real-world effectiveness of these newer regimens would be needed, as these are being widely used [26]. Moreover, liposomal irinotecan (MM-398) in combination with 5-FU and leucovorin has shown efficacy in patients with gemcitabine refractory pancreatic cancer, and was recently approved in the United States [27].

Because the importance of cost-effectiveness has increased in relation to limited healthcare budgets and the disproportionate rise in spending on cancer drugs, we also performed a cost-effectiveness analysis from the perspective of a national health insurance payer. Given the South Korean WTP threshold of USD 27,272, GEM-E was not cost-effective compared to GEM alone. In line with our finding, Tam et al. examined the cost-effectiveness of gemcitabine combinations for metastatic pancreatic cancer and found that GEM-E was not a cost-effective option compared with GEM alone [26]. Furthermore, they proposed FOLFIRNOX might be cost-effective compared to GEM alone if the WTP threshold was relatively high or if drug costs were substantially reduced. Further studies on the cost-effectiveness of recent chemotherapy regimens such as FOLFIRINOX or nab-paclitaxel, in the context of a nation’s WTP background, are warranted.

## Conclusion

In conclusion, this national population-based study did not prove that GEM-E was significantly superior to GEM for pancreatic cancer, providing only a modest difference in survival. The lack of effectiveness and cost effectiveness of GEM-E in a South Korean cohort shown in the present study suggests that reconsideration of the use of GEM-E for pancreatic cancer is warranted and further multinational trials are needed.

## Abbreviations

CCI, charlson comorbidity Index; GEM, gemcitabine; GEM-E, gemcitabine and erlotinib; NHIS, National Health Insurance Service; OS, overall survival; WTP, willingness-to-pay

## Additional file

Additional file 1: Table S1. Hazard ration of survival. (DOCX 16 kb)
