# Supplementary material for: Erlotinib plus gemcitabine versus gemcitabine for pancreatic cancer: real-world analysis of Korean national database
Source: BMC Cancer. 2016 Jul 11;16:443. doi: 10.1186/s12885-016-2482-z (PMC4940912; doi:10.1186/s12885-016-2482-z)
Supplement: Additional file 1: Table S1. — Hazard ration of survival. (DOCX 16 kb) [file 12885_2016_2482_MOESM1_ESM.docx]

Supplementary Table

Table S1. Hazard ration of survival

|  | Hazard Ratio | 95% Hazard Ratio Confidence Interval | | *p*-value^a^ |
| --- | --- | --- | --- | --- |
| GEM | 1 |  |  |  |
| GEM-E | 0.966 | 0.901 | 1.036 | 0.3353 |
| Sex | | | | |
| Female | 1 |  |  |  |
| Male | 1.085 | 1.016 | 1.158 | 0.0151 |
| Age | | | | |
| 18-29 | 1 |  |  |  |
| 30-39 | 1.774 | 0.706 | 4.457 | 0.2226 |
| 40-49 | 2.106 | 0.871 | 5.095 | 0.0983 |
| 50-59 | 2.265 | 0.941 | 5.456 | 0.0682 |
| 60-69 | 2.416 | 1.004 | 5.815 | 0.0490 |
| 70-79 | 3.027 | 1.257 | 7.290 | 0.0135 |
| Above 80 | 3.381 | 1.379 | 8.290 | 0.0078 |
| Charlson Comorbidity Index (CCI) | | | | |
| ≤ 3 | 1 |  |  |  |
| 4-6 | 1.034 | 0.895 | 1.195 | 0.6481 |
| 7-9 | 1.311 | 1.142 | 1.504 | 0.0001 |
| 10-12 | 1.592 | 1.397 | 1.813 | <0.0001 |
| ≥ 13 | 1.767 | 1.527 | 2.044 | <0.0001 |
| Abbreviation: GEM, Gemcitabine; GEM-E, Gemcitabine + Erlotinib  ^a^ chi-square test | | | | |
